# Do health care workforce, population, and service provision significantly contribute to the total health expenditure? An econometric analysis of Serbia

**DOI:** 10.1186/s12960-016-0146-3

**Published:** 2016-08-15

**Authors:** M. Santric-Milicevic, V. Vasic, Z. Terzic-Supic

**Affiliations:** 1 Institute of Social Medicine, Faculty of Medicine, University of Belgrade, Dr Subotica 15, 11000 Belgrade, Serbia; 2 Center School of Public Health and Health Management, Faculty of Medicine, University of Belgrade, Pasterova 2, 11000 Belgrade, Serbia; 3 Department of Statistics and Mathematics, Faculty of Economics, University of Belgrade, Kamenicka 6, 11000 Belgrade, Serbia

**Keywords:** Health care workforce, Population growth, Service provision, Total health expenditure, Econometric analysis, Serbia

## Abstract

**Background:**

In times of austerity, the availability of econometric health knowledge assists policy-makers in understanding and balancing health expenditure with health care plans within fiscal constraints. The objective of this study is to explore whether the health workforce supply of the public health care sector, population number, and utilization of inpatient care significantly contribute to total health expenditure.

**Methods:**

The dependent variable is the total health expenditure (THE) in Serbia from the years 2003 to 2011. The independent variables are the number of health workers employed in the public health care sector, population number, and inpatient care discharges per 100 population. The statistical analyses include the quadratic interpolation method, natural logarithm and differentiation, and multiple linear regression analyses. The level of significance is set at *P* < 0.05.

**Results:**

The regression model captures 90 % of all variations of observed dependent variables (adjusted *R* square), and the model is significant (*P* < 0.001). Total health expenditure increased by 1.21 standard deviations, with an increase in health workforce growth rate by 1 standard deviation. Furthermore, this rate decreased by 1.12 standard deviations, with an increase in (negative) population growth rate by 1 standard deviation. Finally, the growth rate increased by 0.38 standard deviation, with an increase of the growth rate of inpatient care discharges per 100 population by 1 standard deviation (*P* < 0.001).

**Conclusions:**

Study results demonstrate that the government has been making an effort to control strongly health budget growth. Exploring causality relationships between health expenditure and health workforce is important for countries that are trying to consolidate their public health finances and achieve universal health coverage at the same time.

**Electronic supplementary material:**

The online version of this article (doi:10.1186/s12960-016-0146-3) contains supplementary material, which is available to authorized users.

## Background

Health expenditures are one of the fastest-growing elements of public spending in many countries over the past few decades [1, 2]. Based on the Organisation for Economic Co-operation and Development (OECD) data for the 35-year period, the lag between the average growth rate of health spending per capita and growth of gross domestic product (GDP) per capita is rising in many European countries. In the first decade of the twenty-first century, well-developed countries had on average 2.6 % annual growth in GDP per capita and 3.9 % growth per capita in health spending [1]. As of 2007, the Republic of Serbia, which is a middle-income European country, is also facing the mismatch between economic progress (GDP growth rate fell from 3.8 to −1.8 % in 2014 [3]) and health spending (about 10.4 % of GDP [4, 5]). To sustain growth in health spending, the governments of many countries are taking measures toward drivers of health expenditure such as costs for the health workforce, infrastructure, and organization of health care delivery [6].

Drivers of health spending are forces at the demand and the supply side of the framework of the health care provision [7]. They have been analyzed in a numerous cross-sectional and time series studies over the last 50 years [8]. Extensive literature covers the demand-side determinants of health expenditure such as demography (population size, age and sex structure, life expectancy, and healthy life years) and epidemiology characteristics (morbidity, death proximity) and socio-economic context (GDP, living conditions) [2, 7–18]. The most-studied drivers on the supply side are health care policy and institutional framework (resources, financing, insurance, provision services, and products) and technology (diffusion of specific therapy and equipment, information communication technology) [2, 7, 11, 13, 14, 19].

Most of the research was about the correlation between health expenditure and GDP that is considered as one of the main drivers of total health expenditure growth [9, 10, 13]. The cross-sectional econometric analyses of GDP per capita explain 92 % of the differences in the level and growth of total health expenditures [10]. GDP growth rate contributes to health spending variations in low- and middle-income countries while the reverse causality is recorded in high-income countries [11]. However, a few longitudinal studies have found a relationship between GDP and health spending, and that was due to the lack of other reliable variables [7, 20, 21].

The impact of determinants of health spending beyond GDP has been studied more in the past decade [2, 13–15]. Science and technology innovations are altering both the health care provision (health workforce competencies and health care settings) and the customers’ expectations which influence health spending [19]. Several studies have demonstrated that the demand for health care depends on the health status (morbidity, disability) mainly [7, 13, 22]. Health status is described as the transmission mechanism between the age and the consumption of health care [23–26]. In various contexts, an aging population is estimated to contribute to 6.5–9 % of the increase of health spending while medical processes and income 5–18 % and 28–58 %, respectively [13–15]. The impact of technological progress was assessed as the highest (50–75 %) [13]. The medical practice adjusted for morbidity was estimated to explain a quarter of the increase in health spending [14]. Relevant studies have pointed out that more efficient equipment and treatment practices offset the changes in morbidity and can reduce unit costs while they may induce the demand for health care and total health expenditure even more strongly than the effects of the age structure of the population [14, 27]. The empirical evidence on the association between the increase in the health workforce supply and the increase in health expenditure is inconclusive [7, 28]. Compared with pure demographic drivers, the growth of unit costs in the labor-intensive sector, such as health care, is projected to increase (reduce) health expenditure if it grows faster (slower) than the GDP per capita [7].

Given the fact that all of these may influence the efficiency and utilization of health workers, there is a need for more analyses that attempt to explain changes in health workforce budgets, in particular from less-developed countries [12, 29]. The objective of this study is to explore the health workforce supply and inpatient care provision in the public health care sector and population size as drivers of the total health expenditure in Serbia.

### Drivers of health expenditure in the Republic of Serbia

The Republic of Serbia (excluding data for Kosovo and Metohija) is a South Eastern European country with approximately 7 million people. The Serbian population is aging (an average age of 41 years) [30], and non-communicable diseases and injuries are the leading causes of premature mortality [31]. Health risk behavior, such as tobacco and alcohol consumption, has been very frequent among the population for a long time [32]. The country is in the midst of a transition from a centralized to a market-oriented economy. So far, a slow economic transition has led Serbia to high unemployment rates (22 %) and a rather low Human Capital Index score of −0.343 [33].

As in many countries in the world, the Serbian health system has been making efforts to improve the health of the population within its financial capacity. To improve efficiency and utilization of the health system, as of 2002, the health sector of Serbia has undergone significant steps, such as the emergent reconstruction of state hospitals (including reduction of hospital bed numbers), two cycles of cadre rationalization, and decentralization of primary care [34, 35]. The health sector reform comprised the renewal of the legislation and norms and standards for staffing, performance, and quality of health care. Also, clinical guidelines and an integrated health information system were introduced, equipment renewed, and the chambers for health professionals, the Health Council of Serbia, and the accreditation agency were constituted [35].

In the period from 2007 to 2013, total health spending as a percentage of GDP has been constant (from 10.4 to 10.6) [4, 5]. In the same period, more than half of the total public health expenditure has been traditionally spent on salaries, with around 3 % represented by capital investments and 6 % by preventive health services while the inpatient care expenditure was over six times higher [5]. During the past decade, the share of private health expenditure has been rising (mostly for pharmaceuticals), and now, it constitutes 4 % of GDP [5]. Throughout that period, the accessibility of physicians, nurses, and midwives per 10 000 population has increased by 14 % but with significant inequity across districts [36]. The current government has additionally frozen salaries and has introduced selective deployment in the public health care sector and has announced further cadre rationalization though there are a high unemployment rate and strong migration intentions among health professionals [37, 38]. The recent report on health system effectiveness in Serbia suggests focusing on improving the basic functions and solving inequality, corruption, poor access, and the saturation of inpatient care [39].

## Methods

### Study variables and econometric model presentation

This study focus was on three drivers of the total health expenditure (THE) in Serbia from 2003 to 2011. The reason on focusing on a small set of drivers was the fact that valid regression models of the time series data cannot bear many potential explanatory variables [40]. The causality relation between the total health expenditure and GDP is well established by empirical evidence, showing that GDP impact may countermand the impact of other variables. The Serbian health budget (10.4 to 10.6 % of GDP) was under special policy conduct during the economic transition, and the plan is not to change it in the future [5, 41]. This study analyzed the contribution of the number of health workers (physicians, dentists, pharmacologists, nurses, and midwives) employed in the public health care sector (Sum_HW), the number of the population (Population), and the number of all inpatient care discharges per 100 population (SP) to the health expenditure growth in Serbia over the period of 9 years.

Health workers (physicians, dentists, pharmacologists, nurses, and midwives) represent a key structural input in the health system. At the beginning of the studied period, in 2003, the sum of observed health workers represented 85 % of all employees in the Serbian public health care sector. Regardless of its drop to 66 % in 2011 [42], the estimates presented here are broadly applicable to key providers of direct health care services in the country.

The decision to analyze the public health sector and population size is based on the fact that health care services in Serbia are publicly provided for the whole population via compulsory health insurance, and about 3 % of services are provided in the private sector [43]. An alternate approach is to explore the impact of disease or risk behavior on health expenditure [9–14], such as cancer, cerebrovascular diseases, hypertension, and tobacco and alcohol consumption that are prominent in Serbia [16, 17]. However, the long time series of prevalence or incidence data for these diseases and risk factors are not yet available. Moreover, it is less likely to capture the particular and valid effect of changes in the age and gender structure of the population or that of average life expectancy (LE) and healthy life years on the total health expenditure in the analysis of quarterly data sets. Instead, we decide to use population size to approximate the sum of effects of customers’ attributes on the total health expenditure.

The utilization of inpatient care was of interest in this study for three reasons. Firstly, the inpatient care expenditure is six times higher that the outpatient health care expenditure of Serbia; therefore, it is the stronger driver of health expenditure than the outpatient care. Secondly, the health care reform in Serbia aimed at decreasing inefficiency in this labor-extensive sector of health care [34, 35]. Modeled on reforms in other countries [44], efficiency improvement of inpatient care included a renewal of inpatient care technology and reduction of bed supply to increase inpatient occupancy rate, scrutiny of hospital admission indications, encouraging transfer to day surgery, and reducing average length stay, thus altering the inpatient care utilization. Thirdly, the sophisticated and more expensive technology of inpatient care (magnetic resonance imaging*,* positron emission tomography, and computed tomography scan, to name a few) can be also held responsible for altering the health care costs [2, 13–15] and patients’ discharge [45]. Due to the lack of reliable time series data on the diffusion of technological advances in the country, the number of inpatient care discharges is used to depict its possible effects of health spending throughout the observed period.

Data on total health expenditure (sum of public and private spending on health measured in US$) and on population size are taken from the National Health Accounts (NHA) database of Serbia [5]. For consistency reasons, these data are complemented with data on drivers obtained from the online database “Health for all” of the World Health Organization/Europe [4].

There were no high correlations or collinearity between drivers (Additional file 1). The general construct of regression model Eq. 1 is written as follows:

1 $$ \mathrm{T}\mathrm{H}{\mathrm{E}}_t=\mathrm{B}0+\mathrm{B}1\times \mathrm{S}\mathrm{um}\_\mathrm{H}{\mathrm{W}}_{t-4}+\mathrm{B}2\times \mathrm{Populatio}{\mathrm{n}}_{t-4}+\mathrm{B}3\times \mathrm{S}{\mathrm{P}}_t+\mathrm{B}4\times \mathrm{Indicator} $$

Where

- *t* represents a year, and *t* − 4 represents a four-quartile-lagged variable among regressors.
- The Indicator captures unexpected shifts [40] in the THE time series (dummy variable with all zero values except last two observations, in the third and fourth quarter of 2011, with value one).
- Unstandardized coefficients are presented with B0, B1, B3, and B4.

Using cross-correlation analyses [46] between the dependent variable and quantitative predictors (THE and Sum_HW, and THE and Population), the highest correlation is found with the SUM_HW at lag 4 (Additional file 2: Table S1 and Population at lag 0 (Additional file 2: Table S2). More precisely, the ordinary least squares (OLS) equation provides the strong (first order) positive autocorrelation at lag 0 for the variable Population (see Durbin-Watson statistic value in Table 1 in the “Results” section). Hence, we choose to involve the variable Population with lag 4 (based on information that our time series are on quarterly not annual levels). Thus, the positive (first order) autocorrelation disappears (Table 1 in the “Results” section), and with this lag, residuals show good performance (Table 2 in the “Results” section). Between the two stationary time series THE and SP, the maximal correlation with the variable SP is found in no lagged form, i.e., lag 0 (Additional file 2: Table S3).

**Table 1:** Descriptors of the regression model

Model summary^a^						
Dependent variable		*R*	*R* square	Adjusted *R* square	Std. error of the estimate	Durbin-Watson
THE		0.956^b^	0.914	0.901	0.00750	2.148
ANOVA^b^						
Model		Sum of squares	df	Mean square	*F*	Sig.
1	Regression	0.016	4	0.004	69.241	0.000^a^
	Residual	0.001	26	0.000		
	Total	0.017	30			

^a^Predictors: (constant), Indicator, Sum_HW_lag_4, SP, Population_lag_4

^b^Dependent variable: *THE* total health expenditure

**Table 2:** Descriptors of the residuals of the regression model

One-sample Kolmogorov-Smirnov test					
			Unstandardized residual		
*N*			31		
Normal parameters^a,b^		Mean	0.0000000		
		Std. deviation	0.00698488		
Most extreme differences		Absolute	0.143		
		Positive	0.143		
		Negative	−0.143		
Test statistic			0.143		
Asymp. sig. (2-tailed)			0.104^c^		
Autocorrelations of series unstandardized residual					
Lag	Autocorrelation	Std. error^d^	Box-Ljung statistic		
			Value	df	Sig.^e^
1	−0.096	0.171	0.318	1	0.573
2	0.384	0.168	50.527	2	0.063
3	0.024	0.165	50.549	3	0.136
4	0.151	0.162	60.408	4	0.171
5	−0.189	0.159	70.820	5	0.166
6	−0.029	0.156	70.853	6	0.249

^a^Test distribution is normal

^b^Calculated from data

^c^Lilliefors significance correction

^d^The underlying process assumed is independence (white noise)

^e^Based on the asymptotic chi-square approximation

Furthermore, standardized coefficients (beta) are presented that allow for a comparison of the independent variable’s unique contribution to the dependent variable [47], since values of all variables in the model have been converted to the same scale, and their variances are controlled.

The statistical analysis includes the conversion of a frequency of data series from yearly to quarterly frequency. The conversion was done with the Kernel interpolation method (the “quadratic match average” procedure for stock data and the “quadratic match sum” procedure for flow data) with the Econometric and Time Series Software EViews7 (Quantitative Micro Software, LLC) [48]. These two methods fit a local quadratic polynomial for each observation of the low-frequency series and then use this polynomial to fill in all observations of the high-frequency series associated with the period. The quadratic polynomial is formed by taking sets of three adjacent points from the source series and fitting a quadratic so that either the average or the sum of the high-frequency points matches the low-frequency data observed [48]. These quadratic methods were selected from the existing interpolation methods as adequate for the low- to high-frequency variables in our analysis.

In the next step, natural logarithm and differentiation are used to bring all data series to the stationary level (Additional file 3). In this manner, transformed values represent growth rates [49]. Providing the unit root test on all time series on the level and first difference, we conclude that all first difference series in the OLS equation are stationary.

Multiple linear regression analyses are conducted with *t* tests and *F* tests to assess the statistical significance of the regression model and coefficients [50]. The assessment of model adequacy includes testing for the normal distribution of the model residuals (one-sample Kolmogorov-Smirnov (KS) test with Lilliefors significance correction) and autocorrelation (Durbin-Watson statistic and Box-Ljung statistic) [50]. The level of significance is set at *P* < 0.05. These procedures are completed in IBM SPSS Statistics 23 (IBM Corporation) [51].

## Results

### Description of variables

**Fig. 1:**
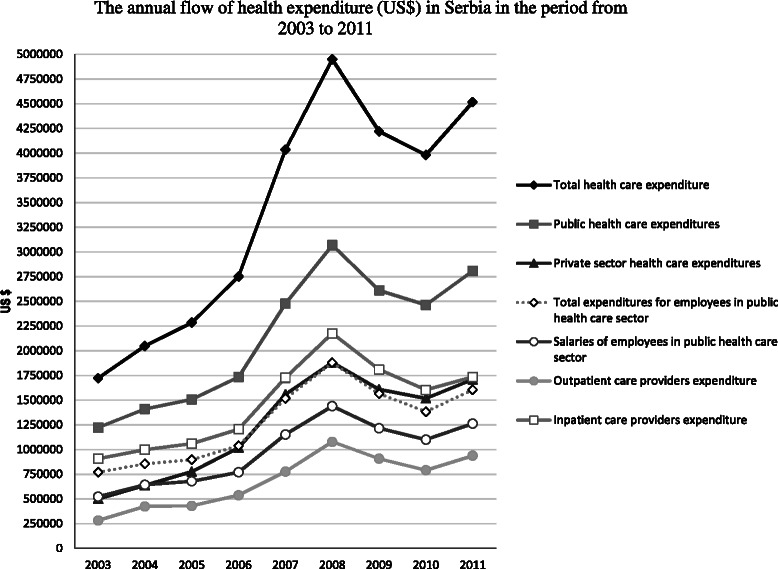
The annual flow of indicators of health expenditure (US$) in Serbia from 2003 to 2011. *Black rhombuses* on the *back line* represent total health care expenditure; *dark gray squares* on the *dark gray line* represent public health care expenditure; *white squares* on the *gray line* represent inpatient care providers expenditures; *white triangles* on the *dotted gray line* represent total expenditures for employees in public health care sector; *dark gray triangles* on the *dark gray line* represent private sector health care expenditure; *white circles* on the *dark gray line* represent salaries for employees in public health care sector; *gray circles* on the *gray line* represent outpatient care providers’ expenditure; *gray crosses* on the *gray line* represent inpatient care providers’ expenditure

Throughout the observed period, total health expenditure (THE) and other indicators of health expenditure had an increasing trend by 2008 and then dropped in successive years and began to recover in 2011 (Fig. 1). The share of public health expenditure decreased from 71 % in 2003 to 62 % in 2011 while the private sector expenditure increased from 29 to 38 % in the respective years (Fig. 1). Though salaries for public health employees rose in the period from 2003 to 2011 (from 68 to 79 % of the total expenditures for public health employees), the total expenditures for public health employees declined (from 45 to 36 % of THE) (Fig. 1). The outpatient care provider expenditure varied, and in the year 2011, it represented 21 % of THE (Fig. 1). The inpatient care provider expenditure decreased from 53 % in 2003 to 38 % of THE in 2011 (Fig. 1).

**Fig. 2:**
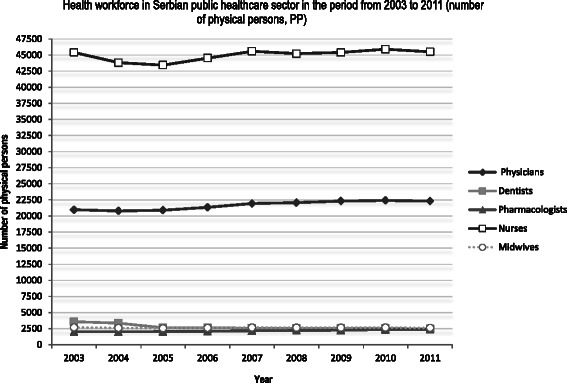
Health workforce in the Serbian public health care sector from 2003 to 2011 (number of physical persons). *White squares* on the *black line* represent nurses; *dark gray rhombuses* represent a number of physicians; *white squares* represent a number of dentists; *gray squares* on the *gray line* represent a number of dentists; *white circles* on the *dotted line* represent a number of midwives; *dark gray triangles* on the *dark gray line* represent a number of pharmacists

While the total number of observed health workers increased, the number of dentists and midwives decreased. The highest increase was registered among physicians (Fig. 2).

**Fig. 3:**
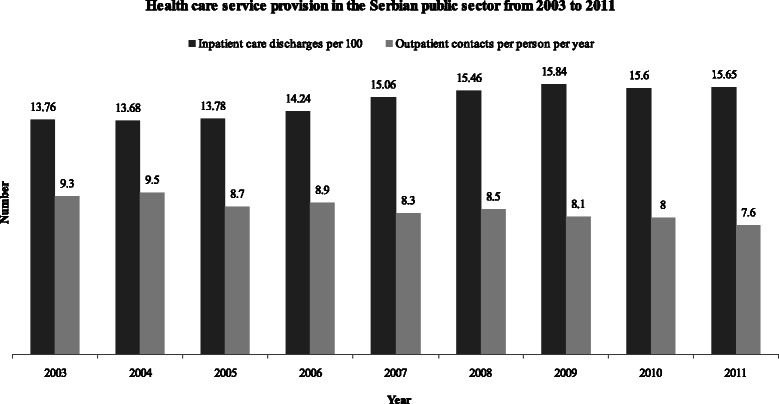
Health care service provision in the Serbian public sector from 2003 to 2011. *Black columns* represent inpatient care discharges per 100 population; *gray columns* represent outpatient contacts per person per year

Inpatient care discharges per 100 population increased from 13.8 in 2003 to 15.7 in 2011, while the number of outpatient contacts per person per year decreased from 9.3 in 2003 to 7.6 in 2011 (Fig. 3).

### Econometric model results

The regression model captures 90 % of all variations of observed dependent variables (adjusted *R* square), and the model is significant (*F* = 39.241; *P* < 0,001 (Table 1)). The Durbin-Watson test for serial correlation with extreme sample sizes or many regressors shows no autocorrelations (Table 1).

Residuals have normal distribution according to the one-sample KS test (*P* = 0.104 (Table 2)). The autocorrelation of residuals assessed with the Box-Ljung statistic shows a value of 0.249; therefore, these residuals represent white noise, i.e., no autocorrelation is identified (Table 2).

The application of the unstandardized coefficients in the regression model (Table 3) leads to the following data in Eq. 1:

2 $$ \mathrm{T}\mathrm{H}{\mathrm{E}}_t=0.128+5.318\times \mathrm{S}\mathrm{um}\_\mathrm{H}{\mathrm{W}}_{t-4}+137.315\times \mathrm{Populatio}{\mathrm{n}}_{t-4}+1.416\times \mathrm{S}{\mathrm{P}}_t+0.046\times \mathrm{Indicator} $$

**Table 3:** Regression model coefficients

	Descriptive statistics			Coefficients^a^				
	Mean	Std. deviation	*N*	Unstandardized coefficients		Standardized coefficients		
Total health expenditure (THE)	0.0130	0.02384	31	*B*	Std. error	Beta	*t*	Sig.
Constant				0.128	0.010		13.053	0.000
The number of health workers Sum_HW_lag_4	0.0001	0.00542	31	5.318	0.381	1.210	13.948	0.000
The number of population Population_lag_4	−0.0009	0.00019	31	137.315	10.863	1.117	12.640	0.000
Inpatient care discharges per 100 population SP	0.0045	0.00635	31	1.416	0.224	0.377	6.324	0.000
Indicator	0.0645	0.24973	31	0.046	0.006	0.479	7.793	0.000

^a^Dependent variable: *THE* total health expenditure

According to the standardized coefficients (Table 3), the model shows a direct and significant relationship between the dependent and independent variables. THE growth increases by 1.21 standard deviations with the increase of health workforce growth rate by 1 standard deviation (*P* < 0.001). However, THE growth rate decreases by 1.12 standard deviations with the increase of the population growth rate (because it is negative; see Table 2) by 1 standard deviation (*P* < 0.001). THE growth rate also increases by 0.38 standard deviation with the increase of the growth rate of inpatient care discharges per 100 population by 1 standard deviation (*P* < 0.001).

## Discussion

The results of this study have identified the short-term causality relationship between total health expenditure and health workers, population number, and inpatient care discharges based on their direct contribution to the total health expenditure.

The growth of the health workers’ number in the previous year strongly contributed to the growth of the total health expenditure as indicated by the standardized coefficients. That effect may be explained by the rise in employment of higher-skilled health workers due to technological diffusion in the public health system [7]. The efforts to attain universal and comprehensive health coverage and better access to quality health services in recent decades may have resulted in increased workforce supply, as well as in the rise of health spending [7].

Study results about the direct and short-term causality between the increase in the growth rate of the health workforce and the increase in the growth rate of total health expenditure are supported by findings of the French project [52] and the 25-year Canadian study [53]. There is a link between the increase in the number of physicians and the increase in health costs in France [52]. The number of physicians per capita has significant positive impact on health expenditure in Canadian provinces [53]. On the contrary, in the pooled cross-sectional analyses, the total expenditure decreases with an increase in the density of physicians per capita [10]. In the private practice, there was no link between the increase in the number of practitioners and the increase in demand [54, 55]. The growth of physicians’ supply had no significant impact on the total health expenditure’s evolution in the 8-year Korean study and the Singapore analysis of drivers of over 40 years [56, 57].

A model with disaggregated data regarding workforce category will most probably yield altered results, and this difference may be important since these categories have a different share in total workforce (nurses and midwives comprise about 65 % of the health workforce in this study) and wage rates. For example, the ratio of net salaries between nurses and midwives at the secondary education level and physicians’ subspecialists was approximately 1 to 2.33 [58].

The contribution of the growth of the population size in the previous year was slightly lower than the contribution of the health workforce. Several studies have found no clear link between health spending and the demographic situation of societies over time [23], but in this study, it caused an increase of 1.12 standard deviations of health expenditure growth rate. There is the agreement that proximity of death and a significant share of ill elderly may increase health spending [7, 13, 16, 22]. Therefore, population contribution is most likely a reflection of the effects of the morbidity and disability [9–14] such as malignant and cerebrovascular diseases that are more prominent in comparison with many countries in the Europe region, particularly in the Europe Union, for which Serbia is a candidate country [4]. About 12 % of LE in Serbia (average LE is 74 years) is estimated to be unhealthy life years [33]. It may also reflect the impact of demographic aging [13–17]. Further potential reason is the impact of depopulation due to negative population growth by 0.5 % annually [30], declining share of young population, decisive fall in fertility [59], and an emigration. The other explanation may be the fact that somewhat higher health expenditure is estimated in countries with publicly provided health care via social health insurance [7] such as in Serbia. The Serbian government is also covering the health care costs of the inactive population (the highest unemployment rate is for population under the age of 25 years [60]) and some other vulnerable groups via taxes while 24.6 % of the population is at risk of poverty (the level of relative poverty is estimated to be approximately 100 Euros per household per month) [60].

The study result about the link between inpatient care provision (indirect impact of medical process organization and technology diffusion) and health expenditure is consistent with the literature [2, 11–13, 19]. The contribution of the growth of the inpatient care utilization in the same year was almost one third less than the contribution of the population. In our study, total health expenditure increased by 0.38 standard deviation with an increase of the growth rate of inpatient care discharges by 1 standard deviation. This effect may be explained by a higher registration of inpatient services (by 13 %) due to the possibly limited provision of curative and screening services or poor medical and technological equipment at the primary health care level [61]. It also reflects the progress of technology in the Serbian inpatient care over the observed period. Therefore, we expected it would have a smaller contribution to health spending growth then the health workforce. If the burden of chronic morbidity worsens or preserves at the same level, the inpatient health expenditure may increase [16, 17]. Also, diffusion of technological innovations may present an economic burden in the longer term [18].

Labor input (human and capital) to health systems are subject to the regulatory and institutional framework drivers. Therefore, a single contribution of observed drivers that caused about 1 or less than 1 standard deviation of the growth rate of total health spending in Serbia can be seen as the government works to control health budget growth while increasing the health workforce availability and inpatient service provision at the same time.

The statistical approach used to assess both the time series model nature and the risk of the model is supported by the literature [40]. This model has captured the dynamics of regressors and unexpected shifts of the THE time series (represented by the Indicator in the model). The unexpected shifts may be the result of some intervention in the third and fourth quartiles of 2011. Those interventions could have been part of the final phase of projects in the health care sector or/and tailored by the election of a new government in the year 2012. The contribution of these structural breaks in the model was included to prevent incorrect conclusions regarding the behavior of time series and to avoid poor forecasts [62].

An exploration of the model (forecast) limitations (Additional file 4) has proven that the significant categorical predictor of the first period of the creation of the OLS model will be lost when splitting the sample period into the last several observations. That will happen since the last significant predictor of THE is the categorical dummy variable (which has a positive value 1 for the last two observations and a value of zero for all others). Therefore, a model risk is presented as well as the model nature.

### Limitations of the study

This study has certain limitations due to a study period of 9 years, which we tried to overcome by conversion of yearly into quarterly data. However, the last available data on THE by January 2015 were for the period from 2003 to 2011. Thus, despite the relevance of the regression model, it could not identify the long-term impact. Another limitation comes from the constraints of the number of variables that can be included in a time-series-based model [7]. With a larger number of potential predictors and due to the structural breaks, the reliability of the time series models and the feasibility of projections decline [7]. Variables used in this study assessed the contribution of broad variables based on aggregated data instead of particular characteristics, as seen in other studies (for example, physicians, and population aged 65+ years).

### Policy implications

Essential and extensive funding of the literature on the drivers of health expenditure comes from well-developed countries, with rare information from less-developed countries [9–11, 53, 63, 64] though their health care systems are also facing challenges in fiscal sustainability. In Serbia, some research on health spending has pointed out the effects of population aging, pharmaceutical market evolution, and out-of-pocket expense growth [65–68]. The recent longitudinal research showed that GDP positively correlates with physician and nurse supply, the population size, and the number of inpatient care discharges in Serbia [69]. In that regard, this study provides potentially valuable information about the impact of policy measures on labor variables that are commonly rationalized in countries under fiscal constraints.

Though relevant for Serbian context, study results are consistent with similar research done in various settings. This econometric model identified the highest single contribution of health worker supply to the increase of total health expenditure. Decreasing health budgets may harm efforts for universal health coverage by worsening waiting lists and inequality in workforce supply and performance. Also, economic benefits of investments in health care measured by health indicators (LE, mortality) would significantly pay off in the future [70]. The results of the study support the disaggregated analysis concerning the health workforce impact in health expenditure growth. Selective decision will allow more efficient steering of the health system while facing fiscal policies within 1.5 % expected real growth of GDP at market price in 2016 [3]. Decision-making about workforce reduction in the public health care sector should consider simulation analyses of the workforce supply disaggregated by age, sex, skill mix, and workplace/region given that workforce categories differ by deployment and retirement dynamics. For example, the number of physicians and secondary-level nurses had positive annual growth (0.2 and 1 %, respectively), while the growth rate of dentists and higher-educated nurses and midwives was negative (−3.7, −1.4, and −2.2 %, respectively) [58]. On the other hand, the expected overall outflow due to retirement in 2017 is approximately 17 % of physicians without a specialization, 23 % of physician specialists, 21 % of dentists, 15 % of pharmacists, and 5 % of nurses and midwives. Higher outflow is expected from outpatient (11 %) than from inpatient health care (8 %) [58].

The regression model has succeeded in capturing the short-term balance between the dynamic variables and should be used for short-term predictions only. The standardized coefficients obtained in this analysis can also be used for other, more practical purposes in the long run if the model is updated. Based on the growth model of the economy in Serbia by 2020, a reduction of the share of public expenditure on health care in GDP in Serbia is less likely in the future than a reallocation of existing expenditures [41]. Due to the fiscal deficit and high public debt, the experts’ opinion is that the health system needs a reform of the current financing system [71–73]. To provide efficient and higher-quality health care and universal coverage, the reform should be based on input and significant rationalization (reducing the number of non-medical staff, the number of hospital beds, etc.).

## Conclusions

The growth of the health workforce number in the previous year has strongly contributed to the growth of total health expenditure in Serbia from 2003 to 2011. The contribution of the growth of the population size in the previous year was slightly lower, and the contribution of the growth of the inpatient care utilization in the same year was almost one third less.

Exploring this type of causality relationship is important for countries that are undertaking policy measures to consolidate public health finances and achieve universal health coverage at the same time.

## Abbreviations

GDP, gross domestic product; KS, Kolmogorov-Smirnov; LE, The life expectancy; NHA, National Health Accounts; OECD, Organisation for Economic Co-operation and Development; OLS, ordinary least squares; SP, the number of all inpatient care discharges per 100 population; Sum_HW, physicians, dentists, pharmacists, nurses, and midwives employed in public health care sector; THE, total health expenditure; US$, United States dollars

## Additional files

Additional file 1: Collinearity between predictors. (DOC 75 kb)

Additional file 2: Cross-correlation analysis and lag of variables. (DOC 80 kb)

Additional file 3: Testing a unit root of the time series. (DOC 240 kb)

Additional file 4: An exploration of the model (forecast) limitations. (DOC 84 kb)
