# Supplementary material for: Do health care workforce, population, and service provision significantly contribute to the total health expenditure? An econometric analysis of Serbia
Source: Hum Resour Health. 2016 Aug 15;14:50. doi: 10.1186/s12960-016-0146-3 (PMC4986341; doi:10.1186/s12960-016-0146-3)
Supplement: Supplementary file 1 — Collinearity between predictors. (DOC 75 kb) [file 12960_2016_146_MOESM1_ESM.doc]

**Additional File 1. Collinearity between predictors**

In our study, predictor "Indicator" isn't correlated with any other predictors. Significance level of Pearson correlation coefficient between predictor "Indicator" and predictor "Sum_HM_lag_4" is 0.454 which is much greater from alfa level 0.05. (table - Correlations).

| **Correlations** | | | | | | |
| --- | --- | --- | --- | --- | --- | --- |
|  | | THE | Sum_HW_lag_4 | Population_lag_4 | Sp | Indicator |
| Pearson Correlation | THE | 1,000 | ,495 | ,056 | ,434 | ,186 |
|  | Sum_HW_lag_4 | ,495 | 1,000 | -,719 | ,262 | -,022 |
|  | Population_lag_4 | ,056 | -,719 | 1,000 | -,219 | -,227 |
|  | Sp | ,434 | ,262 | -,219 | 1,000 | -,034 |
|  | Indicator | ,186 | -,022 | -,227 | -,034 | 1,000 |
| Sig. (1-tailed) | THE | . | ,002 | ,382 | ,007 | ,159 |
|  | Sum_HW_lag_4 | ,002 | . | ,000 | ,077 | ,454 |
|  | Population_lag_4 | ,382 | ,000 | . | ,119 | ,109 |
|  | Sp | ,007 | ,077 | ,119 | . | ,427 |
|  | Indicator | ,159 | ,454 | ,109 | ,427 | . |
| N | THE | 31 | 31 | 31 | 31 | 31 |
|  | Sum_HW_lag_4 | 31 | 31 | 31 | 31 | 31 |
|  | Population_lag_4 | 31 | 31 | 31 | 31 | 31 |
|  | Sp | 31 | 31 | 31 | 31 | 31 |
|  | Indicator | 31 | 31 | 31 | 31 | 31 |

Also, between predictors, we have no problem of high correlations or collinearity (table – Coefficients: Collinearity Statistics, Tolerance and VIF).

| **Coefficients^a^** | | | | | | | | |
| --- | --- | --- | --- | --- | --- | --- | --- | --- |
| Model | | Unstandardized Coefficients | | Standardized Coefficients | t | Sig. | Collinearity Statistics | |
|  |  | B | Std. Error | Beta |  |  | Tolerance | VIF |
| 1 | (Constant) | ,128 | ,010 |  | 13,053 | ,000 |  |  |
|  | Sum_HW_lag_4 | 5,318 | ,381 | 1,210 | 13,948 | ,000 | ,439 | 2,279 |
|  | Population_lag_4 | 137,315 | 10,863 | 1,117 | 12,640 | ,000 | ,423 | 2,365 |
|  | Sp | 1,416 | ,224 | ,377 | 6,324 | ,000 | ,927 | 1,078 |
|  | Indicator | ,046 | ,006 | ,479 | 7,793 | ,000 | ,875 | 1,142 |
| a. Dependent Variable: THE | | | | | | | | |

…or better "Condition Index" in table - Collinearity Diagnostics.

| **Collinearity Diagnostics^a^** | | | | | | | | |
| --- | --- | --- | --- | --- | --- | --- | --- | --- |
| Model | Dimension | Eigenvalue | Condition Index | Variance Proportions | | | | |
|  |  |  |  | (Constant) | Sum_HW_lag_4 | Population_lag_4 | Sp | Indicator |
| 1 | 1 | 2,592 | 1,000 | ,00 | ,00 | ,00 | ,05 | ,02 |
|  | 2 | 1,065 | 1,560 | ,00 | ,28 | ,00 | ,03 | ,19 |
|  | 3 | ,864 | 1,732 | ,00 | ,12 | ,00 | ,02 | ,64 |
|  | 4 | ,469 | 2,351 | ,00 | ,05 | ,00 | ,90 | ,04 |
|  | 5 | ,009 | 16,821 | ,99 | ,55 | ,99 | ,00 | ,11 |
| a. Dependent Variable: THE | | | | | | | | |
