# Supplementary material for: Do health care workforce, population, and service provision significantly contribute to the total health expenditure? An econometric analysis of Serbia
Source: Hum Resour Health. 2016 Aug 15;14:50. doi: 10.1186/s12960-016-0146-3 (PMC4986341; doi:10.1186/s12960-016-0146-3)
Supplement: Supplementary file 2 — Cross-correlation analysis and lag of variables. (DOC 80 kb) [file 12960_2016_146_MOESM2_ESM.doc]

Additional file 2: Cross-correlation analysis and lag of variables

| Date: 10/25/15 Time: 07:09 |  |  |  |  |
| --- | --- | --- | --- | --- |
| Sample: 2003Q1 2011Q4 | |  |  |  |
| Included observations: 35 | |  |  |  |
| Correlations are asymptotically consistent approximations | | | | |
|  |  |  |  |  |
|  |  |  |  |  |
| THE,SUM_HW(-i) | THE,SUM_HW(+i) | i | Lag | lead |
|  |  |  |  |  |
|  |  |  |  |  |
| . | . | | . | . | | 0 | 0.0243 | 0.0243 |
| . |**. | | . | . | | 1 | 0.1570 | 0.0035 |
| . |**. | | . *| . | | 2 | 0.2494 | -0.0418 |
| . |*** | | . *| . | | 3 | 0.3184 | -0.0798 |
| . |***** | | .**| . | | 4 | 0.4785 | -0.1962 |
| . |**. | | . | . | | 5 | 0.2496 | -0.0400 |
| . |* . | | . | . | | 6 | 0.1142 | 0.0381 |
| . *| . | | . |* . | | 7 | -0.0435 | 0.1197 |
|  |  |  |  |  |
|  |  |  |  |  |

| Date: 10/25/15 Time: 07:09 |  |  |  |  |
| --- | --- | --- | --- | --- |
| Sample: 2003Q1 2011Q4 | |  |  |  |
| Included observations: 35 | |  |  |  |
| Correlations are asymptotically consistent approximations | | | | |
|  |  |  |  |  |
|  |  |  |  |  |
| THE,POPULATI(-i) | THE,POPULATI(+i) | i | Lag | lead |
|  |  |  |  |  |
|  |  |  |  |  |
| . *| . | | . *| . | | 0 | -0.1334 | -0.1334 |
| . *| . | | . *| . | | 1 | -0.0873 | -0.0739 |
| . *| . | | . | . | | 2 | -0.0536 | -0.0361 |
| . | . | | . | . | | 3 | -0.0072 | 0.0044 |
| . |* . | | . | . | | 4 | 0.0506 | 0.0392 |
| . |* . | | . |* . | | 5 | 0.1152 | 0.0865 |
| . |**. | | . |* . | | 6 | 0.1820 | 0.1225 |
| . |**. | | . |* . | | 7 | 0.2491 | 0.1487 |
|  |  |  |  |  |
|  |  |  |  |  |

| Date: 10/25/15 Time: 07:12 |  |  |  |  |
| --- | --- | --- | --- | --- |
| Sample: 2003Q1 2011Q4 | |  |  |  |
| Included observations: 35 | |  |  |  |
| Correlations are asymptotically consistent approximations | | | | |
|  |  |  |  |  |
|  |  |  |  |  |
| THE,SP(-i) | THE,SP1(+i) | i | Lag | lead |
|  |  |  |  |  |
|  |  |  |  |  |
| . |**** | | . |**** | | 0 | 0.3887 | 0.3887 |
| . |*** | | . |**** | | 1 | 0.3445 | 0.4436 |
| . |*** | | . |***** | | 2 | 0.2777 | 0.4643 |
| . |**. | | . |***** | | 3 | 0.1988 | 0.4612 |
| . |**. | | . |***** | | 4 | 0.1804 | 0.4879 |
| . *| . | | . |*** | | 5 | -0.0442 | 0.3424 |
| .**| . | | . |**. | | 6 | -0.1972 | 0.2385 |
| ***| . | | . |* . | | 7 | -0.3388 | 0.1283 |
|  |  |  |  |  |
|  |  |  |  |  |

| ***Model Summary^b^*** | | | | | |
| --- | --- | --- | --- | --- | --- |
| *Model* | *R* | *R Square* | *Adjusted R Square* | *Std. Error of the Estimate* | *Durbin-Watson* |
| *1* | *,696^a^* | *,484* | *,405* | *,01840* | *,727* |
| *a. Predictors: (Constant), Indicator, Sum_HW_lag_4, Sp, Population* | | | | | |
| *b. Dependent Variable: THE* | | | | | |

| ***Coefficients^a^*** | | | | | | | | |
| --- | --- | --- | --- | --- | --- | --- | --- | --- |
| *Model* | | *Unstandardized Coefficients* | | *Standardized Coefficients* | *t* | *Sig.* | *Collinearity Statistics* | |
|  |  | *B* | *Std. Error* | *Beta* |  |  | *Tolerance* | *VIF* |
| *1* | *(Constant)* | *,090* | *,038* |  | *2,354* | *,026* |  |  |
|  | *Sum_HW_lag_4* | *3,609* | *1,035* | *,821* | *3,488* | *,002* | *,358* | *2,790* |
|  | *Population* | *86,769* | *39,228* | *,533* | *2,212* | *,036* | *,342* | *2,920* |
|  | *Sp* | *1,131* | *,551* | *,301* | *2,053* | *,050* | *,922* | *1,085* |
|  | *Indicator* | *,036* | *,015* | *,373* | *2,333* | *,028* | *,775* | *1,291* |
| *a. Dependent Variable: THE* | | | | | | | | |
