# Supplementary material for: Do health care workforce, population, and service provision significantly contribute to the total health expenditure? An econometric analysis of Serbia
Source: Hum Resour Health. 2016 Aug 15;14:50. doi: 10.1186/s12960-016-0146-3 (PMC4986341; doi:10.1186/s12960-016-0146-3)
Supplement: Supplementary file 3 — Testing a unit root of the time series. (DOC 240 kb) [file 12960_2016_146_MOESM3_ESM.doc]

Additional file 3: Testing a unit root of the time series

| Null Hypothesis: THE has a unit root | | | |  |
| --- | --- | --- | --- | --- |
| Exogenous: Constant, Linear Trend | | | |  |
| Lag Length: 0 (Automatic - based on SIC, maxlag=8) | | | | |
|  |  |  |  |  |
|  |  |  |  |  |
|  |  |  | t-Statistic | Prob.* |
|  |  |  |  |  |
|  |  |  |  |  |
| Augmented Dickey-Fuller test statistic | | | -2.093834 | 0.5307 |
| Test critical values: | 1% level |  | -4.252879 |  |
|  | 5% level |  | -3.548490 |  |
|  | 10% level |  | -3.207094 |  |
|  |  |  |  |  |
|  |  |  |  |  |
| *MacKinnon (1996) one-sided p-values. | | | |  |
|  |  |  |  |  |
|  |  |  |  |  |
| Augmented Dickey-Fuller Test Equation | | | |  |
| Dependent Variable: D(THE) | | |  |  |
| Method: Least Squares | | |  |  |
| Date: 10/25/15 Time: 06:59 | | |  |  |
| Sample (adjusted): 2003Q3 2011Q4 | | | |  |
| Included observations: 34 after adjustments | | | |  |
|  |  |  |  |  |
|  |  |  |  |  |
| Variable | Coefficient | Std. Error | t-Statistic | Prob. |
|  |  |  |  |  |
|  |  |  |  |  |
| THE(-1) | -0.267751 | 0.127876 | -2.093834 | 0.0445 |
| C | 0.004032 | 0.006538 | 0.616648 | 0.5420 |
| @TREND(2003Q1) | -1.04E-05 | 0.000289 | -0.035959 | 0.9715 |
|  |  |  |  |  |
|  |  |  |  |  |
| R-squared | 0.128513 | Mean dependent var | | 0.000426 |
| Adjusted R-squared | 0.072288 | S.D. dependent var | | 0.016756 |
| S.E. of regression | 0.016139 | Akaike info criterion | | -5.331099 |
| Sum squared resid | 0.008074 | Schwarz criterion | | -5.196420 |
| Log likelihood | 93.62868 | Hannan-Quinn criter. | | -5.285169 |
| F-statistic | 2.285692 | Durbin-Watson stat | | 2.143458 |
| Prob(F-statistic) | 0.118589 |  |  |  |
|  |  |  |  |  |
|  |  |  |  |  |

| Null Hypothesis: D(THE) has a unit root | | | |  |
| --- | --- | --- | --- | --- |
| Exogenous: Constant | | |  |  |
| Lag Length: 0 (Automatic - based on SIC, maxlag=8) | | | | |
|  |  |  |  |  |
|  |  |  |  |  |
|  |  |  | t-Statistic | Prob.* |
|  |  |  |  |  |
|  |  |  |  |  |
| Augmented Dickey-Fuller test statistic | | | -7.014487 | 0.0000 |
| Test critical values: | 1% level |  | -3.646342 |  |
|  | 5% level |  | -2.954021 |  |
|  | 10% level |  | -2.615817 |  |
|  |  |  |  |  |
|  |  |  |  |  |
| *MacKinnon (1996) one-sided p-values. | | | |  |
|  |  |  |  |  |
|  |  |  |  |  |
| Augmented Dickey-Fuller Test Equation | | | |  |
| Dependent Variable: D(THE,2) | | |  |  |
| Method: Least Squares | | |  |  |
| Date: 10/25/15 Time: 07:00 | | |  |  |
| Sample (adjusted): 2003Q4 2011Q4 | | | |  |
| Included observations: 33 after adjustments | | | |  |
|  |  |  |  |  |
|  |  |  |  |  |
| Variable | Coefficient | Std. Error | t-Statistic | Prob. |
|  |  |  |  |  |
|  |  |  |  |  |
| D(THE(-1)) | -1.227842 | 0.175044 | -7.014487 | 0.0000 |
| C | 0.000544 | 0.002930 | 0.185740 | 0.8539 |
|  |  |  |  |  |
|  |  |  |  |  |
| R-squared | 0.613481 | Mean dependent var | | 0.000183 |
| Adjusted R-squared | 0.601013 | S.D. dependent var | | 0.026646 |
| S.E. of regression | 0.016831 | Akaike info criterion | | -5.272483 |
| Sum squared resid | 0.008782 | Schwarz criterion | | -5.181785 |
| Log likelihood | 88.99596 | Hannan-Quinn criter. | | -5.241966 |
| F-statistic | 49.20302 | Durbin-Watson stat | | 1.989785 |
| Prob(F-statistic) | 0.000000 |  |  |  |
|  |  |  |  |  |
|  |  |  |  |  |

| Null Hypothesis: SUM_HW has a unit root | | | |  |
| --- | --- | --- | --- | --- |
| Exogenous: Constant, Linear Trend | | | |  |
| Lag Length: 0 (Automatic - based on SIC, maxlag=8) | | | | |
|  |  |  |  |  |
|  |  |  |  |  |
|  |  |  | t-Statistic | Prob.* |
|  |  |  |  |  |
|  |  |  |  |  |
| Augmented Dickey-Fuller test statistic | | | -1.791850 | 0.6865 |
| Test critical values: | 1% level |  | -4.252879 |  |
|  | 5% level |  | -3.548490 |  |
|  | 10% level |  | -3.207094 |  |
|  |  |  |  |  |
|  |  |  |  |  |
| *MacKinnon (1996) one-sided p-values. | | | |  |
|  |  |  |  |  |
|  |  |  |  |  |
| Augmented Dickey-Fuller Test Equation | | | |  |
| Dependent Variable: D(SUM_HW) | | |  |  |
| Method: Least Squares | | |  |  |
| Date: 10/25/15 Time: 07:01 | | |  |  |
| Sample (adjusted): 2003Q3 2011Q4 | | | |  |
| Included observations: 34 after adjustments | | | |  |
|  |  |  |  |  |
|  |  |  |  |  |
| Variable | Coefficient | Std. Error | t-Statistic | Prob. |
|  |  |  |  |  |
|  |  |  |  |  |
| SUM_HW(-1) | -0.192015 | 0.107160 | -1.791850 | 0.0829 |
| C | 0.000873 | 0.001178 | 0.740979 | 0.4643 |
| @TREND(2003Q1) | -4.27E-05 | 5.66E-05 | -0.754217 | 0.4564 |
|  |  |  |  |  |
|  |  |  |  |  |
| R-squared | 0.145199 | Mean dependent var | | 0.000124 |
| Adjusted R-squared | 0.090051 | S.D. dependent var | | 0.003201 |
| S.E. of regression | 0.003053 | Akaike info criterion | | -8.661132 |
| Sum squared resid | 0.000289 | Schwarz criterion | | -8.526453 |
| Log likelihood | 150.2392 | Hannan-Quinn criter. | | -8.615202 |
| F-statistic | 2.632879 | Durbin-Watson stat | | 2.296924 |
| Prob(F-statistic) | 0.087883 |  |  |  |
|  |  |  |  |  |
|  |  |  |  |  |

| Null Hypothesis: D(SUM_HW) has a unit root | | | |  |
| --- | --- | --- | --- | --- |
| Exogenous: Constant | | |  |  |
| Lag Length: 0 (Automatic - based on SIC, maxlag=8) | | | | |
|  |  |  |  |  |
|  |  |  |  |  |
|  |  |  | t-Statistic | Prob.* |
|  |  |  |  |  |
|  |  |  |  |  |
| Augmented Dickey-Fuller test statistic | | | -6.778595 | 0.0000 |
| Test critical values: | 1% level |  | -3.646342 |  |
|  | 5% level |  | -2.954021 |  |
|  | 10% level |  | -2.615817 |  |
|  |  |  |  |  |
|  |  |  |  |  |
| *MacKinnon (1996) one-sided p-values. | | | |  |
|  |  |  |  |  |
|  |  |  |  |  |
| Augmented Dickey-Fuller Test Equation | | | |  |
| Dependent Variable: D(SUM_HW,2) | | | |  |
| Method: Least Squares | | |  |  |
| Date: 10/25/15 Time: 07:02 | | |  |  |
| Sample (adjusted): 2003Q4 2011Q4 | | | |  |
| Included observations: 33 after adjustments | | | |  |
|  |  |  |  |  |
|  |  |  |  |  |
| Variable | Coefficient | Std. Error | t-Statistic | Prob. |
|  |  |  |  |  |
|  |  |  |  |  |
| D(SUM_HW(-1)) | -1.195998 | 0.176437 | -6.778595 | 0.0000 |
| C | 0.000134 | 0.000564 | 0.238286 | 0.8132 |
|  |  |  |  |  |
|  |  |  |  |  |
| R-squared | 0.597138 | Mean dependent var | | -5.92E-05 |
| Adjusted R-squared | 0.584142 | S.D. dependent var | | 0.005018 |
| S.E. of regression | 0.003236 | Akaike info criterion | | -8.570131 |
| Sum squared resid | 0.000325 | Schwarz criterion | | -8.479434 |
| Log likelihood | 143.4072 | Hannan-Quinn criter. | | -8.539614 |
| F-statistic | 45.94936 | Durbin-Watson stat | | 1.976161 |
| Prob(F-statistic) | 0.000000 |  |  |  |
|  |  |  |  |  |
|  |  |  |  |  |

| Null Hypothesis: POPULATI has a unit root | | | |  |
| --- | --- | --- | --- | --- |
| Exogenous: Constant, Linear Trend | | | |  |
| Lag Length: 0 (Automatic - based on SIC, maxlag=8) | | | | |
|  |  |  |  |  |
|  |  |  |  |  |
|  |  |  | t-Statistic | Prob.* |
|  |  |  |  |  |
|  |  |  |  |  |
| Augmented Dickey-Fuller test statistic | | | -2.010788 | 0.5748 |
| Test critical values: | 1% level |  | -4.252879 |  |
|  | 5% level |  | -3.548490 |  |
|  | 10% level |  | -3.207094 |  |
|  |  |  |  |  |
|  |  |  |  |  |
| *MacKinnon (1996) one-sided p-values. | | | |  |
|  |  |  |  |  |
|  |  |  |  |  |
| Augmented Dickey-Fuller Test Equation | | | |  |
| Dependent Variable: D(POPULATI) | | | |  |
| Method: Least Squares | | |  |  |
| Date: 10/25/15 Time: 07:03 | | |  |  |
| Sample (adjusted): 2003Q3 2011Q4 | | | |  |
| Included observations: 34 after adjustments | | | |  |
|  |  |  |  |  |
|  |  |  |  |  |
| Variable | Coefficient | Std. Error | t-Statistic | Prob. |
|  |  |  |  |  |
|  |  |  |  |  |
| POPULATI(-1) | -0.190267 | 0.094623 | -2.010788 | 0.0531 |
| C | -0.000159 | 6.30E-05 | -2.520718 | 0.0171 |
| @TREND(2003Q1) | -1.97E-06 | 1.85E-06 | -1.066343 | 0.2945 |
|  |  |  |  |  |
|  |  |  |  |  |
| R-squared | 0.143761 | Mean dependent var | | -1.92E-05 |
| Adjusted R-squared | 0.088519 | S.D. dependent var | | 6.19E-05 |
| S.E. of regression | 5.91E-05 | Akaike info criterion | | -16.55083 |
| Sum squared resid | 1.08E-07 | Schwarz criterion | | -16.41615 |
| Log likelihood | 284.3640 | Hannan-Quinn criter. | | -16.50490 |
| F-statistic | 2.602412 | Durbin-Watson stat | | 2.323534 |
| Prob(F-statistic) | 0.090204 |  |  |  |
|  |  |  |  |  |
|  |  |  |  |  |

| Null Hypothesis: D(POPULATI) has a unit root | | | |  |
| --- | --- | --- | --- | --- |
| Exogenous: Constant | | |  |  |
| Lag Length: 0 (Automatic - based on SIC, maxlag=8) | | | | |
|  |  |  |  |  |
|  |  |  |  |  |
|  |  |  | t-Statistic | Prob.* |
|  |  |  |  |  |
|  |  |  |  |  |
| Augmented Dickey-Fuller test statistic | | | -6.874769 | 0.0000 |
| Test critical values: | 1% level |  | -3.646342 |  |
|  | 5% level |  | -2.954021 |  |
|  | 10% level |  | -2.615817 |  |
|  |  |  |  |  |
|  |  |  |  |  |
| *MacKinnon (1996) one-sided p-values. | | | |  |
|  |  |  |  |  |
|  |  |  |  |  |
| Augmented Dickey-Fuller Test Equation | | | |  |
| Dependent Variable: D(POPULATI,2) | | | |  |
| Method: Least Squares | | |  |  |
| Date: 10/25/15 Time: 07:03 | | |  |  |
| Sample (adjusted): 2003Q4 2011Q4 | | | |  |
| Included observations: 33 after adjustments | | | |  |
|  |  |  |  |  |
|  |  |  |  |  |
| Variable | Coefficient | Std. Error | t-Statistic | Prob. |
|  |  |  |  |  |
|  |  |  |  |  |
| D(POPULATI(-1)) | -1.206946 | 0.175562 | -6.874769 | 0.0000 |
| C | -2.28E-05 | 1.14E-05 | -2.002099 | 0.0541 |
|  |  |  |  |  |
|  |  |  |  |  |
| R-squared | 0.603897 | Mean dependent var | | 4.59E-07 |
| Adjusted R-squared | 0.591119 | S.D. dependent var | | 9.76E-05 |
| S.E. of regression | 6.24E-05 | Akaike info criterion | | -16.46657 |
| Sum squared resid | 1.21E-07 | Schwarz criterion | | -16.37588 |
| Log likelihood | 273.6985 | Hannan-Quinn criter. | | -16.43606 |
| F-statistic | 47.26245 | Durbin-Watson stat | | 1.986397 |
| Prob(F-statistic) | 0.000000 |  |  |  |
|  |  |  |  |  |
|  |  |  |  |  |

| Null Hypothesis: SP1 has a unit root | | | |  |
| --- | --- | --- | --- | --- |
| Exogenous: Constant, Linear Trend | | | |  |
| Lag Length: 0 (Automatic - based on SIC, maxlag=8) | | | | |
|  |  |  |  |  |
|  |  |  |  |  |
|  |  |  | t-Statistic | Prob.* |
|  |  |  |  |  |
|  |  |  |  |  |
| Augmented Dickey-Fuller test statistic | | | -2.248121 | 0.4493 |
| Test critical values: | 1% level |  | -4.252879 |  |
|  | 5% level |  | -3.548490 |  |
|  | 10% level |  | -3.207094 |  |
|  |  |  |  |  |
|  |  |  |  |  |
| *MacKinnon (1996) one-sided p-values. | | | |  |
|  |  |  |  |  |
|  |  |  |  |  |
| Augmented Dickey-Fuller Test Equation | | | |  |
| Dependent Variable: D(SP) | | |  |  |
| Method: Least Squares | | |  |  |
| Date: 10/25/15 Time: 07:04 | | |  |  |
| Sample (adjusted): 2003Q3 2011Q4 | | | |  |
| Included observations: 34 after adjustments | | | |  |
|  |  |  |  |  |
|  |  |  |  |  |
| Variable | Coefficient | Std. Error | t-Statistic | Prob. |
|  |  |  |  |  |
|  |  |  |  |  |
| SP (-1) | -0.254326 | 0.113128 | -2.248121 | 0.0318 |
| C | 0.001914 | 0.001608 | 1.190789 | 0.2428 |
| @TREND(2003Q1) | -4.01E-05 | 7.39E-05 | -0.542852 | 0.5911 |
|  |  |  |  |  |
|  |  |  |  |  |
| R-squared | 0.146259 | Mean dependent var | | 0.000240 |
| Adjusted R-squared | 0.091179 | S.D. dependent var | | 0.004434 |
| S.E. of regression | 0.004227 | Akaike info criterion | | -8.010733 |
| Sum squared resid | 0.000554 | Schwarz criterion | | -7.876054 |
| Log likelihood | 139.1825 | Hannan-Quinn criter. | | -7.964804 |
| F-statistic | 2.655398 | Durbin-Watson stat | | 2.240829 |
| Prob(F-statistic) | 0.086209 |  |  |  |
|  |  |  |  |  |
|  |  |  |  |  |

| Null Hypothesis: D(SP1) has a unit root | | | |  |
| --- | --- | --- | --- | --- |
| Exogenous: Constant | | |  |  |
| Lag Length: 0 (Automatic - based on SIC, maxlag=8) | | | | |
|  |  |  |  |  |
|  |  |  |  |  |
|  |  |  | t-Statistic | Prob.* |
|  |  |  |  |  |
|  |  |  |  |  |
| Augmented Dickey-Fuller test statistic | | | -7.110268 | 0.0000 |
| Test critical values: | 1% level |  | -3.646342 |  |
|  | 5% level |  | -2.954021 |  |
|  | 10% level |  | -2.615817 |  |
|  |  |  |  |  |
|  |  |  |  |  |
| *MacKinnon (1996) one-sided p-values. | | | |  |
|  |  |  |  |  |
|  |  |  |  |  |
| Augmented Dickey-Fuller Test Equation | | | |  |
| Dependent Variable: D(SP,2) | | |  |  |
| Method: Least Squares | | |  |  |
| Date: 10/25/15 Time: 07:04 | | |  |  |
| Sample (adjusted): 2003Q4 2011Q4 | | | |  |
| Included observations: 33 after adjustments | | | |  |
|  |  |  |  |  |
|  |  |  |  |  |
| Variable | Coefficient | Std. Error | t-Statistic | Prob. |
|  |  |  |  |  |
|  |  |  |  |  |
| D(SP(-1)) | -1.240181 | 0.174421 | -7.110268 | 0.0000 |
| C | 0.000274 | 0.000774 | 0.353822 | 0.7259 |
|  |  |  |  |  |
|  |  |  |  |  |
| R-squared | 0.619893 | Mean dependent var | | 1.03E-05 |
| Adjusted R-squared | 0.607631 | S.D. dependent var | | 0.007087 |
| S.E. of regression | 0.004439 | Akaike info criterion | | -7.937886 |
| Sum squared resid | 0.000611 | Schwarz criterion | | -7.847189 |
| Log likelihood | 132.9751 | Hannan-Quinn criter. | | -7.907369 |
| F-statistic | 50.55592 | Durbin-Watson stat | | 2.000427 |
| Prob(F-statistic) | 0.000000 |  |  |  |
|  |  |  |  |  |
|  |  |  |  |  |
