# Supplementary material for: Do health care workforce, population, and service provision significantly contribute to the total health expenditure? An econometric analysis of Serbia
Source: Hum Resour Health. 2016 Aug 15;14:50. doi: 10.1186/s12960-016-0146-3 (PMC4986341; doi:10.1186/s12960-016-0146-3)

**Additional file 4. An exploration of the model (forecast) limitations**

At first, we have split dataset into an analysis sample and a validation sample (last two years – eight observations). Then, we have provided residual analysis in validation sample. Residual analysis gives good performance of created model. Residuals have well statistical characteristics (table - Descriptives) and normal distribution (table -Tests of Normality).

| **Case Processing Summary** | | | | | | |
| --- | --- | --- | --- | --- | --- | --- |
|  | Cases | | | | | |
|  | Valid | | Missing | | Total | |
|  | N | Percent | N | Percent | N | Percent |
| Unstandardized Residual | 8 | 100,0% | 0 | 0,0% | 8 | 100,0% |

| **Descriptives** | | | | |
| --- | --- | --- | --- | --- |
|  | | | Statistic | Std. Error |
| Unstandardized Residual | Mean | | ,0198526 | ,00792870 |
|  | 95% Confidence Interval for Mean | Lower Bound | ,0011043 |  |
|  |  | Upper Bound | ,0386010 |  |
|  | 5% Trimmed Mean | | ,0192509 |  |
|  | Median | | ,0134190 |  |
|  | Variance | | ,001 |  |
|  | Std. Deviation | | ,02242575 |  |
|  | Minimum | | -,00589 |  |
|  | Maximum | | ,05643 |  |
|  | Range | | ,06232 |  |
|  | Interquartile Range | | ,04156 |  |
|  | Skewness | | ,592 | ,752 |
|  | Kurtosis | | -,963 | 1,481 |

| **Tests of Normality** | | | | | | |
| --- | --- | --- | --- | --- | --- | --- |
|  | Kolmogorov-Smirnov^a^ | | | Shapiro-Wilk | | |
|  | Statistic | df | Sig. | Statistic | df | Sig. |
| Unstandardized Residual | ,201 | 8 | ,200^*^ | ,931 | 8 | ,525 |
| *. This is a lower bound of the true significance. | | | | | | |
| a. Lilliefors Significance Correction | | | | | | |

Residuals are also non-autocorrelated (table – Autocorrelations; figures - ACF and Partial ACF).

| **Autocorrelations** | | | | | |
| --- | --- | --- | --- | --- | --- |
| Series: Unstandardized Residual | | | | | |
| Lag | Autocorrelation | Std. Error^a^ | Box-Ljung Statistic | | |
|  |  |  | Value | df | Sig.^b^ |
| 1 | ,429 | ,296 | 2,107 | 1 | ,147 |
| 2 | ,091 | ,274 | 2,217 | 2 | ,330 |
| 3 | -,178 | ,250 | 2,726 | 3 | ,436 |
| a. The underlying process assumed is independence (white noise). | | | | | |
| b. Based on the asymptotic chi-square approximation. | | | | | |


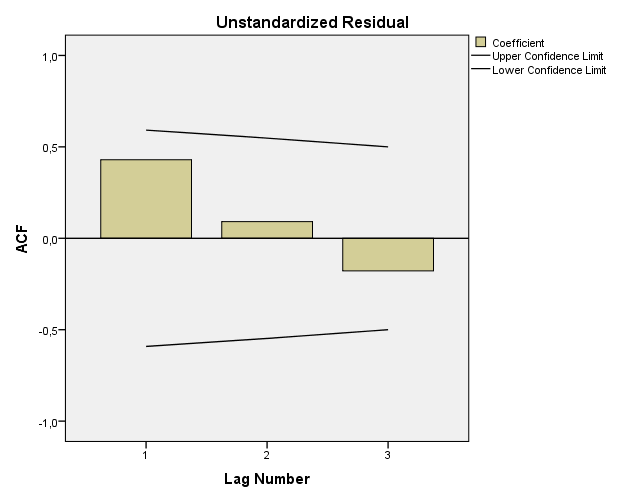


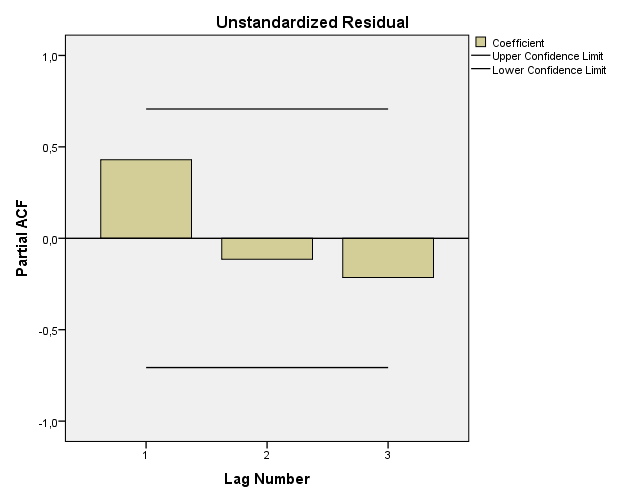

Supplement: Supplementary file 4 — An exploration of the model (forecast) limitations. (DOC 84 kb) [file 12960_2016_146_MOESM4_ESM.doc]
